# Response to Letter to the Editor

**DOI:** 10.1002/clc.24202

**Published:** 2023-12-19

**Authors:** Omar M. Aldaas, Praneet S. Mylavarapu, Jonathan C. Hsu

**Affiliations:** ^1^ Division of Cardiac Electrophysiology at the University of California San Diego Health System La Jolla California USA

To the editor,

We thank Kataoka et al. for their comments regarding our recently published article on catheter ablation of atrial fibrillation in very elderly patients (≥80 years old).^1^ While the unadjusted time‐to‐event analysis may be more reflective of real‐world clinical practice, we adjusted for multiple potential confounders to more clearly assess the association of catheter ablation with all‐cause hospitalizations and mortality in the very elderly. All of the patients included in the study underwent radiofrequency ablation. Although not specifically addressed in our paper, catheter ablation of atrial fibrillation with cryoballoon ablation has also been shown to be safe and effective in the very elderly.^2^, ^3^ We agree that quality of life and mental status before and after ablation is an important outcome that needs to be evaluated further, but this was not collected as part of the UC San Diego AF Ablation Registry. Ultimately, more studies should include the very elderly, as this is a growing population that may benefit from catheter ablation.
